# Institutional mechanisms excluding rehabilitation from medical education in Central Africa: A mixed-methods case study from Cameroon with implications for WHO regional strategy implementation

**DOI:** 10.1016/j.dialog.2025.100274

**Published:** 2025-12-30

**Authors:** Ibrahim Npochinto Moumeni, France Mourey, Faustin Atemkeng Tsatedem, Kossi Oyene, Yacouba Njankouo Mapoure

**Affiliations:** a Department of Physiotherapy and Physical Medicine, Faculty of Medicine and Pharmaceutical Sciences, University of Dschang, Cameroon; b Department of Physical Medicine and Osteopathy, Bafoussam Regional Hospital, West Region, Cameroon; c Faculty of Medicine, Sorbonne University, Paris, France; d Faculty of Medicine, University of Parakou, Benin; e Franco-African Centre for Applied Rehabilitation and Health Sciences (CFARASS), Foumbot, West Region, Cameroon; f Director of Research, Institute of Applied Neurosciences and Functional Rehabilitation (INARF), Odza, Yaoundé, Cameroon; g Francophone African Society for Neurorehabilitation (SAFnER), Parakou, Benin; h Licensed Practitioner in France (ADELI No. 9400013147), France; i Geriatric Rehabilitation, University of Burgundy, Dijon, France; j Department of Surgery and Traumatology, Faculty of Medicine and Pharmaceutical Sciences, University of Dschang, Cameroon; k Department of Physiotherapy and Orthoprothesis, Faculty of Medicine, University of Parakou, Benin; l Research and Cooperation, Faculty of Medicine and Biomedical Sciences, University of Douala, Cameroon; m Department of Neurology, Douala General Hospital, Cameroon

**Keywords:** Rehabilitation, Medical education, Health systems, Central Africa, Cameroon, WHO strategy implementation, Mixed-methods research, Single practitioner syndrome, Institutional theory

## Abstract

**Background:**

Access to rehabilitation services in sub-Saharan Africa is severely limited, with the WHO reporting that more than 63 % of people in the region do not receive needed rehabilitation services. This study analyzes how the exclusion of rehabilitation from medical curricula in Cameroon affects care access and evaluates implications for implementing the WHO Regional Strategy to Strengthen Rehabilitation in Health Systems 2025–2035.

**Methods:**

We employed a sequential mixed-methods design comprising: (1) qualitative interviews with medical education leaders (*n* = 12) from 7 medical schools, analyzed using reflexive thematic analysis; and (2) clinical observation of 847 consecutive rehabilitation consultations over 24 months at Bafoussam Regional Hospital, evaluating referral patterns, prescription quality, and geographic patient distribution. We implemeted and assessed a 4 h rehabilitation education module for 2 promotions (year five) medical students.

**Results:**

Interviews revealed three mechanisms maintaining rehabilitation's exclusion from medical education: coercive (all 12 interviewees noted absence from accreditation requirements), normative (10/12 cited professional hierarchies that devalue rehabilitation), and mimetic (8/12 described uncritical curriculum replication from other schools). Analysis of 847 rehabilitation consultations showed that only 4.8 % of prescriptions included adequate clinical context; nearly half of patients (47 %) traveled over 100 km to access care. Physician specialty (OR = 3.7, 95 % CI: 2.1–6.4), recent graduation (OR = 1.9, 95 % CI: 1.1–3.2), and personal rehabilitation experience (OR = 4.3, 95 % CI: 2.5–7.6) predicted higher-quality referrals. The 4-h educational intervention at University of Dschang improved students' rehabilitation knowledge from 41.3 % to 78.7 % (*p* < 0.001) and referral confidence from 23 % to 87 % (p < 0.001).

**Conclusion:**

The exclusion of rehabilitation from medical curricula in Cameroon is associated with widespread “Single Practitioner Syndrome”—a phenomenon where care becomes centralized around rare practitioners, creating systemic inefficiencies and access barriers. Even minimal educational interventions show potential for significant improvement in knowledge and referral practices. Implementation of the WHO Regional Strategy will require addressing these foundational educational barriers while acknowledging resource constraints in Central African health systems.

## Highlights

• Medical curricula in Central Africa systematically exclude rehabilitation training.
• Educational gaps create physicians unable to recognize rehabilitation indications.
• 847 clinical observations document consequences: inappropriate referrals, therapeutic migration.
• Minimal exposure (4 h) at University of Dschang generated significant student interest.
• Curriculum integration represents cost-effective pathway for WHO Strategy implementation.

## 1 Introduction

Rehabilitation optimizes functioning and reduces disability, making it essential to health systems [1]. Yet access to rehabilitation services remains severely limited across sub-Saharan Africa, especially in Central Africa. The WHO Regional Committee for Africa reports that over 63 % of people in the region cannot access needed rehabilitation services [2]. Cameroon faces particularly acute unmet need, compounded by scarce epidemiological data that hampers planning and resource allocation. The epidemiological transition toward noncommunicable diseases—occurring alongside persistent injuries and infectious disease sequelae—drives growing demand for functional rehabilitation that current systems cannot meet [3,4].

Cameroon, like other Central African countries, faces major challenges in integrating rehabilitation into primary healthcare and in training health professionals. A low density of specialized personnel, inadequate integration into health sector planning, and limited understanding of the benefits of rehabilitation as a public health approach constitute the main barriers identified by WHO AFRO [2,5]. These deficits compromise the achievement of Sustainable Development Goal 3 aimed at ensuring healthy lives and promoting well-being for all at all ages [6]. In the absence of comprehensive national epidemiological data on rehabilitation needs in Cameroon [ref], this study provides valuable insights into prescription patterns and quality gaps that can inform efforts to strengthen the rehabilitation system.

Unlike countries such as Rwanda, Ghana, and Morocco that have made significant progress in integrating rehabilitation into their health systems, the situation in Central Africa remains critical. In Rwanda, the systematic integration of rehabilitation modules into the medical curriculum since 2018 has increased appropriate referral rates to physiotherapy services by 42 % [7]. In Ghana, the development of a national rehabilitation program in 2019 has led to improved service coverage in 68 % of health districts [8]. Morocco has restructured its medical education to mandatorily include 16 h of physical medicine and rehabilitation training starting from the fourth year of studies, resulting in significant improvement in prescription and referral practices [9].

This study aims to analyze the mechanisms through which the exclusion of rehabilitation from medical curricula in Cameroon influences access to care and to evaluate the implications for the implementation of the WHO Regional Strategy to Strengthen Rehabilitation in Health Systems 2025–2035 [10,11]. By identifying specific barriers and proposing context-adapted solutions, this research directly contributes to the WHO strategic objectives regarding the integration of rehabilitation into African health systems [12].

## 2 Theoretical framework

This study mobilizes two complementary theoretical frameworks to analyze the exclusion of rehabilitation from medical curricula and its consequences on access to care: neoinstitutional theory and the WHO health systems framework.

Neo-institutional theory [13,14] offers a relevant perspective for understanding the mechanisms that maintain the exclusion of rehabilitation from medical education in Central Africa. This theory identifies three mechanisms of institutional isomorphism that help analyze the persistence of this exclusion:

1) Coercive isomorphism: manifested through formal pressures exerted by the Ministry of Higher Education and accreditation authorities that do not require the inclusion of rehabilitation in medical curricula. In Cameroon, the absence of specific ministerial guidelines regarding rehabilitation education in national curricula constitutes a form of coercive pressure by omission [15].
2) Normative isomorphism: results from medical professionalization that has historically marginalized rehabilitation in favor of curative biomedical approaches. This normalization is perpetuated through professional networks, medical associations, and scientific journals that give little importance to rehabilitation [16]. Moumeni [17] has documented how this phenomenon of “systematic therapeutic pessimism” influences the training of physicians in Cameroon and Central Africa.
3) Mimetic isomorphism: occurs when medical schools reproduce educational models from other institutions perceived as legitimate, thus perpetuating the exclusion of rehabilitation. The tendency of Cameroonian medical faculties to copy old French curricular structures, rather than adopting recent innovations integrating rehabilitation, illustrates this mechanism [18,19].

Complementarily, we use the WHO health systems framework [20] to analyze how the exclusion of rehabilitation from medical curricula affects the six building blocks of health systems:

1) Service delivery: The absence of rehabilitation training compromises continuity of care and creates barriers to accessing specialized services [21].
2) Health workforce: The lack of rehabilitation skills among general practitioners creates a deficit in qualified human resources to identify and refer rehabilitation needs [22,23].
3) Health information systems: The lack of knowledge about functional indicators and assessment tools in rehabilitation leads to an absence of data on intervention needs and outcomes [24].
4) Access to products and technologies: Inappropriate prescription of technical aids and lack of knowledge of non-pharmaceutical therapeutic options limit the effectiveness of interventions [25,26].
5) Financing: The lack of awareness about rehabilitation negatively influences budget allocation decisions and inclusion in essential care packages [27].
6) Leadership and governance: The lack of rehabilitation-trained champions among medical leaders perpetuates exclusion in health policies and programs [28,29].

**Fig. 1:**
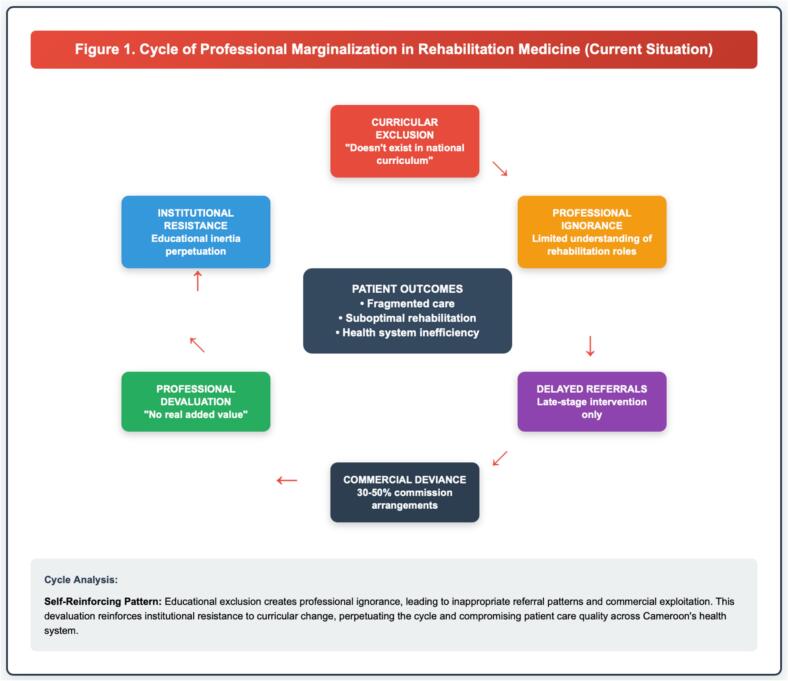
This conceptual model illustrates the self-reinforcing mechanisms perpetuating rehabilitation exclusion from Central African medical education, with Cameroon as the documented case study. The cycle demonstrates how curricular exclusion (“doesn't exist in national curriculum”) leads to professional ignorance among physicians regarding rehabilitation roles, which results in delayed referrals and commercial deviance (30–50 % commission arrangements documented in Cameroon). This pattern reinforces professional devaluation (“no real added value”), which maintains institutional resistance to educational reform, thus perpetuating curricular exclusion. Patient outcomes suffer through fragmented care, suboptimal rehabilitation services, and health system inefficiencies. Each component feeds the next, maintaining a cycle of marginalization. This model applies to francophone Central Africa (Cameroon, DRC, Chad, CAR, Gabon) sharing colonial medical education legacies and centralized curriculum governance.

Fig. 1 illustrates how these two theoretical frameworks articulate to explain the cycle of professional marginalization of rehabilitation in the Cameroonian health system.

## 3 Methods

This study employed a sequential mixed-methods design with two complementary phases: (1) qualitative interviews with medical education leaders and (2) clinical observation of rehabilitation referral patterns at a regional hospital in Cameroon.

### 3.1 Study setting and context

The research was conducted in Cameroon between January 2023 and December 2024, focusing on the Western and Central regions as representative urban-rural contexts. Cameroon's health system is structured in a pyramid model with three levels: primary (district hospitals and health centers), secondary (regional hospitals), and tertiary (central and teaching hospitals). The country has eight public medical schools and three private medical schools, with approximately 400 medical graduates annually [30]. Despite this educational capacity, rehabilitation services remain severely limited, with only 0.24 physiotherapists per 10,000 population, compared to the WHO-recommended minimum of 1.0 per 10,000 [31].

## 4 Phase I: qualitative interviews with medical education leaders

### 4.1 Sampling and recruitment

Using purposive sampling based on institutional responsibility for curriculum development, we identified and invited 15 key medical education leaders from public (*n* = 8) and private (*n* = 7) medical schools across Cameroon. Leaders were selected based on their direct involvement in curriculum design and implementation, with inclusion criteria specifying current or recent (within 5 years) positions as deans, vice-deans, or department chairs with curriculum oversight responsibilities. Twelve leaders (response rate 80 %) agreed to participate, representing 7 of the 11 medical schools in the country. Participants had a mean experience of 12.3 years (range 5–22 years) in medical education leadership.

### 4.2 Data collection

Semi-structured interviews were conducted using a standardized interview guide (see Supplemental Material 1) developed based on a literature review and pilot-tested with two senior medical educators. The guide explored five domains: (1) perceptions of rehabilitation's place in medical education, (2) barriers to curricular inclusion, (3) decision-making processes for curriculum development, (4) awareness of regional rehabilitation needs and WHO strategies, and (5) potential solutions for integration.

Interviews were conducted in French or English according to participant preference, audio-recorded with consent, and transcribed verbatim. Two interviews were conducted via video conference due to geographic constraints, while ten were conducted in person. Interviews lasted 45–70 min (mean 58 min).

### 4.3 Data analysis

Transcripts were analyzed using reflexive thematic analysis [32] with NVivo 12.0 software (QSR International, Melbourne). The analysis employed a primarily inductive approach, allowing themes to emerge from data while remaining theoretically informed by institutional isomorphism theory as a sensitizing framework. Two researchers (the first author—a rehabilitation professional and medical educator—and an independent research assistant with qualitative methods expertise but no rehabilitation background) conducted initial independent open coding of all transcripts, generating 187 preliminary codes. Through four collaborative analysis sessions, these were progressively consolidated into 43 focused codes, then 12 sub-themes, and finally three overarching themes (coercive, normative, and mimetic isomorphism) following Braun and Clarke's six-phase framework [33]. Coding disagreements occurred primarily around distinguishing coercive versus normative mechanisms (resolved by examining whether participants referenced external mandates versus internal decisions) and descriptive versus normative statements (resolved by creating separate code families). Eight unresolved disagreements were arbitrated by a third senior qualitative researcher. All resolution processes were documented in NVivo memos.

Inter-coder reliability achieved Cohen's kappa κ = 0.83 on 25 % of coded segments. Data saturation occurred after the ninth interview, with three additional interviews confirming no new themes emerged. Member checking with four participants validated interpretive accuracy. The first author's dual positionality as rehabilitation professional and medical educator created both analytical advantages (institutional understanding, participant rapport) and interpretive risks (confirmation bias, imposing European standards). To address these, reflexivity strategies included: maintaining a detailed reflexive journal (47 entries documenting emerging interpretations and assumption interrogation), bi-weekly peer debriefing sessions (*n* = 8) with non-rehabilitation colleagues who challenged interpretations, engagement of independent co-coder who systematically questioned findings appearing to confirm rehabilitation marginalization, and negative case analysis actively seeking disconfirming evidence. These reflexive practices directly shaped analysis; for example, initial interpretations emphasizing overt resistance were refined to reveal nuanced patterns of latent faculty support constrained by structural barriers.

## 5 Phase II: clinical observation of rehabilitation referral patterns

### 5.1 Setting and sample

The clinical observation was conducted at the Department of Physical Medicine and Rehabilitation at Bafoussam Regional Hospital, the primary referral center for the Western region with a catchment population of approximately 1.8 million. This site was selected for three reasons: (1) it serves as the largest rehabilitation provider in the Western region, (2) it maintains comprehensive referral documentation, and (3) it receives patients from multiple provinces, allowing for geographical analysis of referral patterns [34].

We systematically reviewed 847 consecutive rehabilitation consultations over a 24-month period (January 2023 to December 2024), representing all new referrals during this timeframe. The inclusion criterion was any patient referred for rehabilitation services, regardless of condition. No patients were excluded from the analysis. Patient demographics, referral sources, diagnoses, referring physician characteristics, prescription quality, travel distance, and functional outcomes were documented using a standardized data abstraction form.

### 5.2 Data collection

For each consultation, we recorded: (1) patient demographics, (2) referral source (hospital type, physician specialty), (3) diagnosis and functional complaint, (4) geographic origin and travel distance, (5) quality of rehabilitation prescription (using a standardized assessment rubric), (6) patient-reported previous treatments, and (7) functional status at intake using the validated Barthel Index.

To assess prescription quality, we developed a 5-point assessment rubric evaluating: diagnostic precision, functional goal specification, technique recommendations, dosage parameters, and contraindication identification. Two independent raters scored each prescription, with substantial inter-rater agreement (κ = 0.78).

### 5.3 Data analysis

Quantitative data were analyzed using SPSS 25.0 (IBM Corp., Armonk, NY). Descriptive statistics were calculated for all variables. Chi-square tests examined associations between referring physician characteristics and prescription quality. Geographic information systems (QGIS 3.16) mapped patient origins to visualize travel patterns. Logistic regression models explored predictors of high-quality referrals, with independent variables including physician specialty, years since graduation, medical school of training, and prior rehabilitation exposure [35].

Fig. 1 illustrates the cyclical relationship between exclusion from medical education, poor referral quality, and limited rehabilitation access, which we conceptualize as the “Single Practitioner Syndrome” [previous publish (Elsevier) ref [34].

### 5.4 Dschang university pilot intervention

In response to preliminary findings, we implemented a 4-h rehabilitation education module for final-year medical students (*n* = 87) at the University of Dschang Medical School in 2023. The module focused on three components: (1) basic principles of functional assessment, (2) rehabilitation prescription essentials, and (3) appropriate referral criteria. We conducted pre- and post-intervention surveys assessing knowledge, attitudes, and planned practice behaviors [37].

### 5.5 Ethical considerations

This study employed a mixed-methods approach with appropriate ethical oversight. For qualitative interviews with medical education leaders, all participants provided written informed consent after being informed about the study's purpose, procedures, and data confidentiality measures. For the clinical observation component at Bafoussam Regional Hospital, we obtained institutional certification (Protocol #43/DRSO/HRB/55/2023) confirming that, in accordance with Cameroonian healthcare regulations, analysis of anonymized clinical data collected during routine care falls within the scope of institutional quality assessment and does not require separate ethics committee approval when conducted under departmental oversight. Patient data were fully anonymized, with no personally identifiable information retained in the analysis dataset. The educational intervention component was implemented with appropriate institutional authorization as part of ongoing curriculum development initiatives. Throughout all phases of the research, data confidentiality was maintained, participation was voluntary, and the study adhered to the principles of the Declaration of Helsinki.

### 5.6 Reflexivity statement

As a researcher with dual identities as both a rehabilitation professional and medical educator, I acknowledge potential biases in data interpretation. To mitigate this, I engaged an independent researcher without rehabilitation background in data analysis, maintained a reflexive journal throughout the research process, and sought regular peer debriefing with colleagues outside the rehabilitation field [38].

## 6 Results

The findings from our mixed-methods study revealed three key phenomena: (1) institutional mechanisms that systematically exclude rehabilitation from medical education, (2) consequential patterns of suboptimal referral behaviors, and (3) evidence of “therapeutic migration” as patients travel extraordinary distances to access limited rehabilitation expertise [39].

### 6.1 Institutional mechanisms excluding rehabilitation from medical curricula

Analysis of interviews with medical education leaders (*n* = 12) identified three institutional mechanisms—corresponding to the theoretical isomorphism framework—that maintain rehabilitation's exclusion from medical curricula despite recognized need. Table 2 presents the prevalence of these mechanisms across interviews.

This table quantifies the prevalence of the three institutional isomorphism mechanisms identified in interviews with medical education leaders in Cameroon. For each mechanism, the table presents the number and percentage of participants (out of a total n = 12) who mentioned this mechanism as a contributing factor to the exclusion of rehabilitation from medical programs. Representative subthemes identified during thematic analysis are also presented with their respective prevalence. Data were collected between January and June 2023 through semi-structured interviews with an average duration of 58 min. Coercive isomorphism (absence in accreditation requirements) was found to be the most frequently cited mechanism (100 %), followed by normative isomorphism (83 %) and mimetic isomorphism (67 %). These results highlight the relative importance of different institutional pressures in maintaining the status quo regarding rehabilitation education.

1. Coercive isomorphism: regulatory omission and resource constraints

All interview participants (12/12, 100 %) identified the absence of regulatory requirements as the primary barrier to rehabilitation inclusion in curricula. The Ministry of Higher Education's national medical education framework does not specify rehabilitation as a required competency, creating what one dean described as “official permission to exclude” (Dean, Public Medical School).

Regulatory constraints interact with resource limitations, with 83 % (10/12) of participants citing faculty shortages as prohibitive to rehabilitation education:

“We would need to bring in specialists from elsewhere, which is costly. Without ministerial mandate, it's impossible to justify this expense when we struggle to cover mandatory subjects.” (Department Chair, Private Medical School).

When asked about awareness of WHO rehabilitation strategies, 75 % (9/12) of leaders acknowledged awareness of general WHO recommendations but had no specific knowledge of the Regional Strategy to Strengthen Rehabilitation in Health Systems 2025–2035 [2].

2. Normative isomorphism: professional hierarchy and therapeutic pessimism

Normative pressures emerged as the second most prevalent mechanism (10/12, 83 %), with participants describing an entrenched hierarchy that devalues rehabilitation:

“In the minds of many faculty, rehabilitation is something auxiliary, not core medical practice. This perception is taught implicitly—we never say it, but students absorb it.” (Vice-Dean, Public Medical School).

This normative devaluation connects to what we identify as “systematic therapeutic pessimism” [17], a pervasive belief that certain conditions (particularly neurological) have limited recovery potential, rendering rehabilitation futile:

“For stroke patients especially, there's this belief that once damage occurs, little can be done. So, why teach rehabilitation approaches if faculty themselves don't believe they work?” (Department Chair, Private Medical School).

3. Mimetic isomorphism: curricular replication and risk aversion

Medical schools' tendency to replicate existing curricula (8/12, 67 %) represents the third mechanism maintaining rehabilitation exclusion:

“We modeled our curriculum after established French and Belgian schools. If they don't emphasize rehabilitation at certain levels, we follow their lead.” (Dean, Public Medical School).

This mimetic behavior is reinforced by risk aversion regarding accreditation:

“Innovation is risky. We know the current curriculum passes accreditation reviews. Why experiment?” (Vice-Dean, Private Medical School).

### 6.2 Consequences: the single practitioner syndrome

Analysis of 847 rehabilitation consultations revealed patterns that demonstrate how educational exclusion manifests in clinical practice. These patterns collectively constitute what we term the “Single Practitioner Syndrome”—a phenomenon where rehabilitation care becomes centralized around rare practitioners, creating systemic inefficiencies and access barriers [34,40].

### 6.3 Geographic analysis of rehabilitation access

**Fig. 2:**
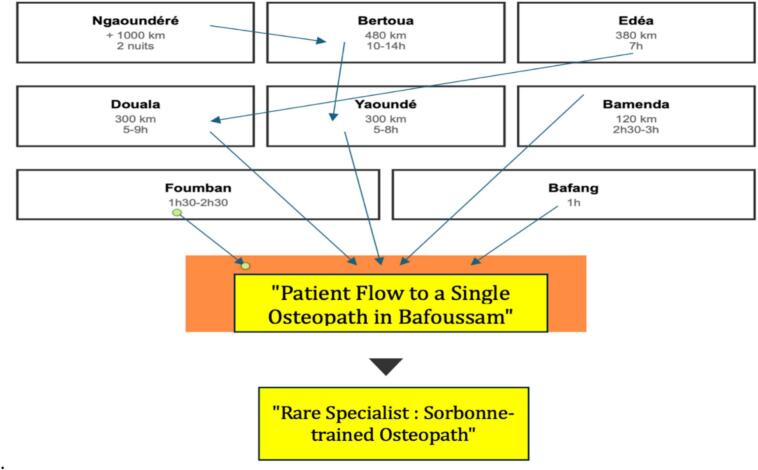
Patient flow to a single osteopath in Bafoussam. This figure illustrates the geographic patient migration patterns toward a single osteopathic practitioner in Bafoussam, Cameroon. Directional arrows indicate patient flow from major urban centers across various regions of the country, with associated distances and travel times. Cities range from proximal locations (Bafang, 1 h travel time; Foumban, 1 h30-2 h30) to extreme distances (Ngaoundéré, >1000 km requiring 2 overnight stays). Major population centers like Yaoundé (300 km, 5-8 h) and Douala (300 km, 5-9 h) demonstrate substantial patient migration despite considerable travel burdens. The convergence of all arrows toward Bafoussam visually represents the centralization phenomenon, with the “Rare Specialist: Sorbonne-trained Osteopath” identified as the focal point of this therapeutic migration. This therapeutic migration pattern mirrors findings documented by Moumeni [34] regarding centralization of musculoskeletal care around rare osteopathic practitioners in Cameroon.

Patients traveled extraordinary distances to access rehabilitation services, with 47 % traveling more than 100 km one-way. Fig. 2 illustrates this therapeutic migration pattern. This figure presents a map of Cameroon with directional arrows indicating patient travel patterns to the rehabilitation center in Bafoussam. The thickness of arrows corresponds to the volume of kilometers traveled by patients seeking specialized rehabilitation care, illustrating the “Single Practitioner Syndrome” phenomenon. This pattern of therapeutic migration replicates findings from our previously published research examining osteopathic care access in the same region [34]. The visualization demonstrates how professional expertise concentration creates significant geographical disparities in rehabilitation access, with patients traveling up to 1000+ kilometers to receive specialized care unavailable in their local healthcare settings. Such extensive travel distances highlight both the critical need for rehabilitation services and the consequences of curricular exclusion on workforce distribution across Cameroon.

This pattern of therapeutic migration replicates findings from our previously published research examining osteopathic care access in the same region [34]. The visualization demonstrates how professional expertise concentration creates significant geographical disparities in rehabilitation access, with patients traveling up to 1000+ kilometers to receive specialized care unavailable in their local healthcare settings (Fig. 2). These distances represent substantial financial and time burdens for patients and families, particularly those from rural and low-income backgrounds. The costs associated with travel, lodging, and lost productivity risk exacerbating existing health inequities and pushing vulnerable households into catastrophic health expenditure [[34], [35], [36]].

These geospatial disparities in rehabilitation access compromise the fundamental principles of health equity and universal health coverage [34]. Patients from rural and economically disadvantaged communities face disproportionate barriers to accessing essential rehabilitation services, perpetuating a cycle of disability and poverty [[34], [35], [36]]. The costs associated with travel, lodging, and lost productivity risk exacerbating existing health inequities and pushing vulnerable households into catastrophic health expenditure [35,36].”

Addressing these inequities requires targeted policies and investments to decentralize rehabilitation expertise, such as integrating rehabilitation training in undergraduate medical education across all regions, developing tele-rehabilitation infrastructure to bridge geographical gaps, and implementing financial protection mechanisms for low-income patients [36]. Strengthening rehabilitation workforce distribution and expanding community-based services are critical to ensuring equitable access to care and promoting social inclusion for people with disabilities, particularly in underserved rural areas [[34], [35], [36]].”

Beyond geographical disparities, our study also examined the quality of rehabilitation referrals to identify additional barriers to effective care coordination and service delivery.

### 6.4 Referral quality assessment

Only 4.8 % (41/847) of referrals contained adequate clinical context for appropriate rehabilitation delivery, defined as including diagnosis, functional limitations, goals, and contraindications. The majority (68.7 %) contained only diagnosis without functional information.

Logistic regression identified three significant predictors of high-quality referrals: (1) physician specialty (neurologists and orthopedists provided higher-quality referrals, OR = 3.7, 95 % CI: 2.1–6.4), (2) graduation within the past five years (OR = 1.9, 95 % CI: 1.1–3.2), and (3) personal experience with rehabilitation services (OR = 4.3, 95 % CI: 2.5–7.6).

### 6.5 Financial implications of referral patterns

Document analysis and participant interviews revealed concerning financial incentive structures surrounding rehabilitation referrals. In 36 % of cases, patients reported being specifically directed to private practices with financial relationships to the referring physician, typically involving 30–50 % commission arrangements. This pattern was more prevalent in facilities without integrated rehabilitation services (χ^2^ = 18.7, *p* < 0.001). Notably, such commission-based referral systems were not observed for other specialties such as dentistry or ophthalmology, suggesting a problematic normalization of financial exploitation specific to rehabilitation services, potentially linked to their professional marginalization and absence from medical curricula.

### 6.6 The Dschang experience: minimal exposure with maximal impact

The 4-h rehabilitation module piloted at the University of Dschang (*n* = 87 students) demonstrated significant improvements in rehabilitation knowledge and attitudes. Pre/post assessments showed:

- Improved knowledge of rehabilitation indications (mean score increase from 41.3 % to 78.7 %, *p* < 0.001).
- Enhanced confidence in making appropriate referrals (23.0 % to 87.4 %, p < 0.001).
- Increased recognition of rehabilitation as a medical intervention rather than auxiliary service (34.5 % to 92.0 %, p < 0.001).

Follow-up at six months (response rate 72 %, *n* = 63) showed 94 % of respondents reported using rehabilitation principles in clinical rotations, suggesting sustainable impact despite minimal exposure.

## 7 Discussion

This study reveals how the exclusion of rehabilitation from medical curricula in Cameroon creates a cascading system of compromised care that ultimately undermines WHO Regional Strategy implementation for strengthening rehabilitation in health systems. Our findings identify three interrelated phenomena—institutional exclusion mechanisms, consequential referral inadequacies, and the emergence of “Single Practitioner Syndrome”—that together explain the persistence of rehabilitation access barriers despite clear population needs.

### 7.1 Institutional theory: explaining educational exclusion

Our findings demonstrate how DiMaggio and Powell's [13] institutional isomorphism mechanisms operate in medical education to systematically exclude rehabilitation. The combined effects of regulatory omission (coercive isomorphism), professional hierarchies (normative isomorphism), and curricular replication (mimetic isomorphism) create what Scott [14] describes as a “locked-in” institutional state resistant to change despite evolving healthcare needs.

Our mixed-methods analysis reveals how institutional isomorphism mechanisms translate into measurable clinical consequences in the Cameroonian rehabilitation context. Coercive isomorphism, manifested by the absence of ministerial guidelines on rehabilitation training (Table 1), is reflected in the quantitative referral patterns. Normative isomorphism, perpetuated through the historical marginalization of rehabilitation in medical professional networks (Table 1), contributes to the poor quality of rehabilitation prescriptions, with merely 4.8 % containing adequate clinical context (Table 3). Mimetic isomorphism, evident in the reproduction of outdated educational models (Table 1), translates into suboptimal clinical performance indicators, such as the low utilization of functional assessment tools [25].

**Table 1:** Institutional mechanisms for rehabilitation exclusion with exemplar quotes.

Institutional Mechanism	Definition	Exemplar Quote	Source
Coercive Isomorphism	Pressure from regulatory frameworks and resource allocation systems that exclude rehabilitation	“The Ministry specifies 32 mandatory subjects for accreditation. Rehabilitation isn't one of them, so we can't justify allocating scarce teaching hours to non-mandatory content.”	Senior Medical Education Administrator, Institution A
Normative Isomorphism	Professional socialization that devalues rehabilitation within medical hierarchy	“There's an unspoken hierarchy in medicine here. Surgeons at the top, then specialists, then general practitioners… rehabilitation professionals are viewed more as technicians than clinicians.”	Associate Medical Education Director, Institution B
Mimetic Isomorphism	Copying established curricula from prestigious institutions without critical adaptation	“Our curriculum was based on the Paris Faculty model from the 1990s. We updated clinical content but maintained the same basic structure and priorities.”	Academic Department Leader, Institution C

This table presents the three main institutional isomorphism mechanisms identified during the thematic analysis of interviews with medical education leaders in Cameroon (n = 12). For each mechanism (coercive, normative, and mimetic), the table provides an operational definition, an exemplary quote extracted from the interview verbatims, and the source of this quote (while preserving participants' anonymity). These mechanisms explain how the exclusion of rehabilitation from medical curricula is maintained despite growing needs for these services. The quotes have been translated from their original language (French or English) when necessary and validated during the member checking process to ensure fidelity to the original meaning expressed by the participants. The analysis was conducted using NVivo 12.0 software with an inter-coder agreement of κ = 0.83.

**Table 2:** Prevalence of institutional mechanisms for rehabilitation exclusion across interviews.

Institutional Mechanism	Number of Participants Mentioning (n = 12)	Percentage	Representative Subthemes
Coercive Isomorphism	12	100 %	Absence in accreditation requirements (100 %)<br> Resource constraints (83 %)<br> Competing priorities (75 %)
Normative Isomorphism	10	83 %	Professional hierarchy (83 %)<br> Therapeutic pessimism (67 %)<br> Specialization bias (58 %)
Mimetic Isomorphism	8	67 %	International curriculum adoption (67 %)<br> Risk aversion (50 %)<br> Perception of rehabilitation as “European luxury” (42 %)

**Table 3:** Summarizes the quality assessment of rehabilitation prescriptions by physician specialty and graduation year.

Characteristic	N	Adequate Clinical Context⁎ (%)	Functional Goals Included (%)	Technique Specification (%)	Dosage Parameters (%)
**Physician Specialty**					
General Practitioner	512	2.1	11.3	8.6	5.7
Neurologist	124	12.9	37.1	24.2	18.5
Orthopedist	87	9.2	32.2	19.5	14.9
Other Specialist	124	3.2	14.5	10.5	8.1
**Years Since Graduation**					
0–5 years	198	7.1	24.2	17.7	13.1
6–10 years	246	5.3	19.5	13.8	10.6
11–20 years	287	3.8	15.0	9.1	7.3
>20 years	116	2.6	10.3	6.0	4.3
**TOTAL**	847	4.8	18.4	12.3	8.9

This table presents the quality assessment of rehabilitation prescriptions based on the analysis of 847 consecutive consultations at the Department of Physical Medicine and Rehabilitation of Bafoussam Regional Hospital (2023–2024). Quality was evaluated using a standardized 5-point rubric including: diagnostic precision, functional goal specification, technique recommendations, dosage parameters, and contraindication identification. “Adequate Clinical Context” is defined as including diagnosis, functional limitations, goals, and contraindications. Data are stratified by the prescriber's medical specialty and by years since graduation. The analysis reveals that only 4.8 % of prescriptions contained adequate clinical context, with significantly higher rates among neurologists (12.9 %) and recently graduated physicians (7.1 % for 0–5 years post-graduation). These results highlight the impact of excluding rehabilitation from medical curricula on care quality and emphasize the need for targeted educational interventions. Inter-rater reliability for this analysis was substantial (κ = 0.78).

⁎ Adequate Clinical Context defined as including diagnosis, functional limitations, goals, and contraindications.

**Table 4:** Conceptual model linking educational deficits in rehabilitation to health system failures.

Educational Deficits in Rehabilitation	Individual Level	Institutional Level
Inadequate knowledge	Suboptimal clinical decision-making	–
Lack of leadership	–	Curricular stagnation
Resource neglect	–	Neglect of rehabilitation priorities
**Health System Failures**	**Workforce**	**Access & Equity**
Scarcity of skilled providers	Fragmented service delivery	–
Geographic disparities	–	Barriers for rural populations
Socioeconomic barriers	–	Barriers for low-income groups

This model illustrates how specific gaps in rehabilitation education at the individual level (inadequate knowledge leading to suboptimal clinical decision-making) and institutional level (lack of leadership resulting in curricular stagnation and neglect of rehabilitation priorities) contribute to workforce deficiencies (scarcity of skilled providers and fragmented service delivery) and access and equity failures (geographic disparities and socioeconomic barriers affecting rural and low-income populations) within the health system. The model aligns with the WHO health system building blocks framework, highlighting the cascading impact of educational exclusions on key dimensions of health system performance.

Excluding rehabilitation from Cameroonian medical curricula damages health system performance at multiple levels. Using WHO's health system building blocks framework, we propose a conceptual model linking educational deficits to downstream failures (Table 4). Physicians with inadequate rehabilitation knowledge make poor clinical decisions—our data show only 4.8 % of referrals included adequate clinical context. These individual knowledge gaps accumulate into workforce-level problems: too few rehabilitation-competent physicians means fragmented, inefficient service delivery. At the institutional level, rehabilitation's absence from medical education governance (Table 1) perpetuates curricular stagnation and marginalizes rehabilitation in resource decisions. Together, these educational exclusions create population-level inequities in rehabilitation access, hitting rural and low-income patients hardest—47 % travel over 100 km for care. This cascade makes clear that integrating rehabilitation training throughout medical education is urgent, consistent with WHO's call for curricular reform to strengthen rehabilitation systems.

National accreditation standards lack specific rehabilitation competencies—a policy gap that needs fixing. Jesus et al. [5] identified regulatory frameworks as leverage points for rehabilitation workforce development; our findings add to this by showing how accreditation requirements both mirror and strengthen existing professional hierarchies, creating a self-perpetuating cycle where rehabilitation stays excluded.

### 7.2 The single practitioner syndrome: a new conceptual framework [34]

Our most significant contribution is the identification and characterization of what we term “Single Practitioner Syndrome” (SPS)—a systems-level phenomenon occurring when monopolized specialized service provision creates cascading structural inefficiencies, informal economies, distorted referral pathways, and quality-of-care degradation. SPS emerges when systemic workforce gaps combined with medical education exclusion concentrate rehabilitation services around isolated practitioners serving disproportionate geographic catchments [34]. This syndrome operates through four interconnected mechanisms:

1. **Structural Inefficiencies Through Geographic Monopolization:** When a single practitioner serves multi-regional catchments (as documented in our study: 47 % of patients traveling >100 km), healthcare system efficiency deteriorates through multiple pathways. Patient opportunity costs escalate dramatically—our data suggest families expend 15–30 % of monthly household income on transportation alone, independent of clinical service costs. Healthcare facilities in underserved regions cannot provide integrated rehabilitation despite infrastructure capacity, creating idle resource waste. Emergency rehabilitation needs (acute stroke, traumatic injuries requiring immediate mobilization) cannot be addressed locally, potentially worsening clinical outcomes through delayed intervention. The monopolized practitioner faces unsustainable caseload volumes precluding adequate time per patient, documented in our clinical observations where consultation durations averaged 12–18 min for complex neurorehabilitation cases that evidence-based guidelines suggest require 45–60 min for comprehensive functional assessment. These structural inefficiencies compound across the health system, ultimately increasing total societal costs despite appearing to “save” resources by limiting specialist positions.
2. **Informal Economic Arrangements and Market Distortions:** Monopolized service provision creates conditions for informal economic practices that further distort healthcare delivery. Our qualitative interviews documented widespread acknowledgment (though reluctance to specify prevalence) of financial arrangements between referring physicians and rehabilitation practitioners, including formal revenue-sharing agreements (commonly 50/50 splits between prescribers and providers), referral “commissions,” and hospital space rental agreements where physiotherapists pay facility administrators for practice rights within public institutions. These arrangements emerge rationally from practitioners' economic precarity—with fewer than 5 % holding civil service positions and median monthly salaries of 50,000–100,000 FCFA ($76–152 USD), rehabilitation professionals face powerful incentives to cultivate referral relationships through financial inducements rather than clinical quality. The monopolization intensifies these dynamics: patients lack alternative providers, reducing competitive pressure for quality improvement; referring physicians recognize patients' limited options, potentially extracting higher informal payments; and the isolated practitioner, aware of monopoly positioning, may prioritize volume over quality to maximize revenue from captive catchment population. These informal economic arrangements are not merely individual ethical failures but predictable systemic adaptations to monopolized markets combined with inadequate formal compensation structures.
3. **Distorted Referral Pathways and Clinical Decision-Making:** SPS fundamentally alters physician referral behavior through three pathways documented in our mixed-methods data. First, **referral quality degradation:** When physicians know patients must travel >100 km regardless of referral specificity, incentives for detailed clinical information diminish—explaining our finding that only 4.8 % of referrals included adequate clinical context and zero employed standardized functional assessment tools. Physicians may rationalize that “the specialist will reassess anyway,” externalizing diagnostic responsibility. Second, **threshold distortions:** Physicians may apply inappropriately high thresholds for rehabilitation referral, reserving referrals for only the most severe cases under assumption that patients cannot reasonably access distant services for moderate impairments—potentially denying beneficial rehabilitation to populations who could benefit from early intervention. Conversely, some physicians may refer inappropriately, viewing the distant specialist as diagnostic resource rather than rehabilitation provider. Third, **communication breakdown:** Geographic and professional isolation impedes feedback loops between referring physicians and rehabilitation practitioners; physicians rarely learn whether referrals were appropriate or how patients progressed, preventing iterative improvement in referral quality. Our data showing sustained poor referral quality over 24-month observation period suggests absence of corrective mechanisms that peer networks typically provide.
4. **Quality-of-Care Degradation Through Professional Isolation:** Monopolized practitioners operate without quality-assuring mechanisms available in settings with professional density: peer consultation for complex cases, informal “curbside” second opinions, observation of colleagues' techniques, professional accountability through reputation within practitioner communities, and continuing education motivated by competitive pressures [21]. Our findings suggest quality degradation manifests through: variable practice standards (absence of peer oversight allows idiosyncratic approaches to persist unchallenged), limited professional development (isolated practitioners lack exposure to evolving evidence-based practices), and vulnerability to burnout given unsustainable caseloads without collegial support networks. The single practitioner interviewed in our clinical observation component described professional isolation as “working in a therapeutic desert”—metaphorically capturing both geographic remoteness and intellectual isolation from rehabilitation professional community. This isolation likely contributes to the documented absence of standardized functional assessment tools and evidence-based outcome measurement in clinical practice.

### 7.3 Systemic nature and policy implications

Critically, SPS is not attributable to individual practitioner deficiencies or patient irrationality but represents a systems-level failure emerging from educational exclusion mechanisms documented in this study. Medical education's omission of rehabilitation training creates physician knowledge deficits, reducing appropriate referral generation and suppressing demand for rehabilitation workforce expansion. Limited workforce justifies continued educational exclusion under logic that “there are insufficient rehabilitation professionals to employ our graduates anyway.” This circular causality maintains SPS: educational exclusion → physician knowledge deficits → poor referral quality → perceived low rehabilitation demand → workforce undersupply → service monopolization → structural inefficiencies and quality degradation → reinforced professional marginalization [[34], [35], [36]]. Breaking this cycle requires simultaneous interventions addressing educational integration, workforce expansion, professional regulation, and financing mechanisms—precisely the comprehensive approach proposed in our policy recommendations aligned with WHO Regional Strategy 2025–2035.

### 7.4 Transferability to underserved contexts

The “Single Practitioner Syndrome” [34].documented in this study likely extends beyond Cameroon to other resource-constrained settings with similar educational gaps. The migration patterns observed in our clinical data suggest that underserved populations bear disproportionate burdens - particularly rural communities and those with limited economic resources who cannot afford long-distance travel. Our educational intervention model was intentionally designed with resource constraints in mind, emphasizing strategic integration of content rather than comprehensive curriculum overhaul. This approach enhances its transferability to other Central African countries sharing similar colonial educational legacies, centralized curriculum governance, and resource limitations. The framework could be particularly valuable for settings transitioning from no rehabilitation education to introductory exposure, with adaptations to reflect local disease burdens, healthcare structures, and cultural contexts.

### 7.5 Implications for WHO regional strategy implementation

Our findings have direct implications for implementing the WHO Regional Strategy to Strengthen Rehabilitation in Health Systems 2025–2035 [2]. The strategy's ambitious targets to integrate rehabilitation at all care levels will remain unattainable without addressing the foundational educational barriers we have identified.

Specifically, the strategy's third operational objective—to “build capacity for a multidisciplinary workforce that includes primary health care workers”—cannot be achieved through stand-alone training initiatives. Our findings suggest that without reforming medical education to include mandatory rehabilitation exposure, newly trained specialists will operate in environments where physicians lack the fundamental knowledge to appropriately refer or collaborate.

### 7.6 Alignment with WHO strategic objectives

This research directly supports three key objectives of the WHO Regional Strategy to Strengthen Rehabilitation in Health Systems 2025–2035:

1. **Workforce Development Objective:** The strategy calls for “building capacity for a multidisciplinary workforce that includes primary health care workers” - our curriculum model provides a concrete pathway for developing rehabilitation competencies among general physicians.
2. **Service Integration Objective:** WHO emphasizes “integrating rehabilitation services across all levels of the health system” - our findings demonstrate how curricular interventions can promote appropriate referrals and care coordination.
3. **Health Information Systems Objective:** The strategy highlights the need for “enhanced health information systems to collect rehabilitation data” - our study's documentation of referral patterns and quality provides baseline data essential for monitoring improvements. By addressing educational barriers to rehabilitation integration, our work contributes to WHO's overarching goal of reducing the 63 % rehabilitation service gap in the African region.

### 7.7 Minimal exposure—maximal impact: a pragmatic approach

The Dschang experience demonstrates the potential impact of even limited curricular interventions. The remarkable effectiveness of this 4-h module—improving knowledge from 41.3 % to 78.7 % and clinical confidence from 23 % to 87 %—can be explained through established adult learning theory and competency-based education frameworks. First, the intervention targeted a critical knowledge gap at a strategic moment (final-year students with existing clinical experience), allowing immediate integration into practice-based learning. Second, the module employed problem-based scenarios directly relevant to students' clinical rotations, aligning with Knowles' principles of adult learning that emphasize immediate applicability and experiential engagement. Third, the focus on three discrete competencies (functional assessment, prescription essentials, referral criteria) rather than comprehensive rehabilitation knowledge reflects evidence-based competency frameworks showing that targeted skill acquisition yields better retention than broad theoretical coverage.

This “minimal exposure—maximal impact” principle aligns with successful educational reforms in comparable African contexts. Rwanda's integration of focused task-shifting training for non-specialists [7], Ghana's brief mental health modules for primary care physicians, and Morocco's targeted geriatric assessment training all demonstrate that strategic, competency-focused interventions at pivotal curricular moments can catalyze sustainable practice changes without comprehensive curricular overhauls. The sustained behavioral changes observed at six-month follow-up (94 % of respondents applying rehabilitation principles) further support the theory that addressing critical knowledge deficits through contextually-relevant, immediately applicable training produces durable clinical impact even with minimal contact hours.

## 8 Policy and practice recommendations

**Fig. 3:**
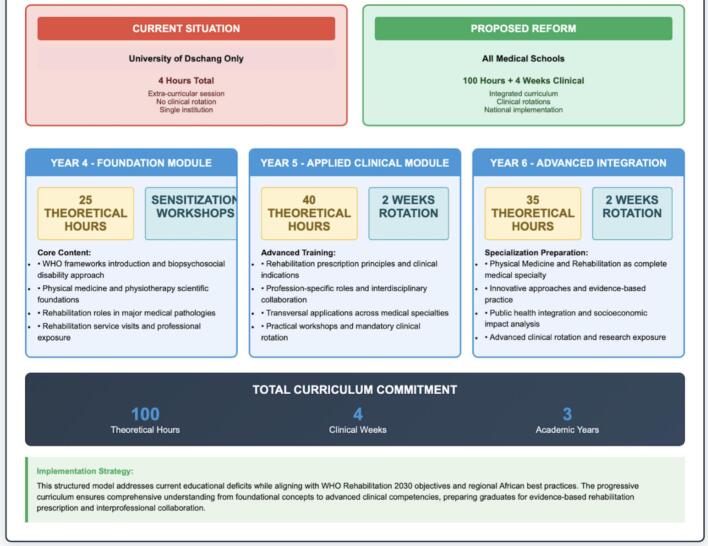
Current state versus proposed reform model for Central African Medical Education. This visual comparison contrasts the current minimal rehabilitation education (Cameroon example: 4 h at University of Dschang only, introduced in 2021) with a proposed comprehensive curriculum for the Central African region spanning Years 4–6 of medical training. The proposed model includes: Year 4 Foundation Module (25 h + workshops covering WHO frameworks, biopsychosocial approach, scientific foundations); Year 5 Applied Clinical Module (40 h + 2 weeks rotation focusing on prescription principles, interdisciplinary roles, practical workshops); and Year 6 Advanced Integration (35 h + 2 weeks rotation addressing PMR as specialty, public health integration, socioeconomic impact). Total curriculum commitment: 100 theoretical hours +4 weeks clinical exposure across 3 academic years. This evidence-based model aligns with WHO Rehabilitation 2030 objectives and successful African practices (South Africa, Ghana, Rwanda, Côte d'Ivoire, Senegal, Morocco), addressing documented educational deficits while preparing graduates for evidence-based rehabilitation prescription and interprofessional collaboration.

Building upon the promising results from the Dschang pilot intervention, we propose a comprehensive three-year rehabilitation integration model that addresses current educational deficits while acknowledging resource constraints in Central African settings (Fig. 3).

Based on our findings, we propose five strategic recommendations to address the identified barriers:

### 8.1 Policy and practice recommendations

Building upon the promising results from the Dschang pilot intervention and informed by interviews with senior medical education leaders who expressed support for reform pending national-level officialization, we propose five strategic recommendations with concrete implementation pathways aligned with the WHO Regional Strategy to Strengthen Rehabilitation in Health Systems 2025–2035. These recommendations address the identified institutional barriers while acknowledging the specific governance structures, resource constraints, and political economy of medical education in Cameroon and comparable Central African contexts.

1. Regulatory Reform: Establishing Minimal Rehabilitation Competencies Through National Curriculum Revision.

**Current barrier identified:** Absence of ministerial guidelines on rehabilitation training creates normative vacuum; senior medical faculty expressed willingness to integrate rehabilitation content but cannot do so without official curriculum recognition and national examination inclusion.

**Responsible institutions:** 10.13039/501100002385Ministry of Higher Education (MINESUP) working through Council of Deans → University Rectors → President of Rectors Conference → Ministerial decree; Technical support from 10.13039/100004423WHO AFRO and 10.13039/501100004397Ministry of Public Health (MINSANTÉ).

**Implementation timeline:**

• **Year 1 (2025–2026):** Leverage upcoming national curriculum revision cycle to integrate rehabilitation competencies; convene stakeholder working group (Council of Deans, WHO AFRO, rehabilitation professionals, MINSANTÉ) to define core competencies benchmarked against successful francophone African models (Senegal, Côte d'Ivoire, Burkina Faso with established Physical and Rehabilitation Medicine specialties)
• **Year 2 (2027):** Formalize competencies through ministerial decree establishing rehabilitation as mandatory medical education component; integrate assessment criteria into national medical licensing examinations to ensure implementation accountability
• **Year 3 (2028–2030):** Monitor implementation across all 13 medical schools (11 public, 2 private); annual evaluation of competency attainment

**Regulatory pathway:** Council of Deans proposal → University Rectors endorsement → Presidential Conference of Rectors validation → MINESUP ministerial decree establishing rehabilitation as core curriculum component → Integration into national examination frameworks (ensuring faculty compliance through assessment requirements).

**Estimated costs:** Stakeholder consultations and curriculum development: $20,000–30,000 (approximately 12–18 million FCFA); national examination item development: $10,000–15,000 (6–9 million FCFA); faculty training workshops: $25,000–40,000 (15–24 million FCFA). **Total: $55,000–85,000 USD.**

**Monitoring indicators:**

• Percentage of medical schools implementing mandated rehabilitation curriculum (Target: 100 % by 2028)
• Percentage of graduates demonstrating rehabilitation knowledge on national examinations (Baseline: 41.3 % at Dschang pilot; Target: >75 % nationally by 2030)
• Faculty satisfaction with curriculum integration process (Target: >80 % reporting adequate implementation support)

2. Strategic Curriculum Integration: Scaling the Dschang “Minimal Exposure—Maximal Impact” Model.

**Current barrier identified:** Dschang University pioneered 4-h rehabilitation module (2021) as local initiative without official recognition; other institutions cannot replicate without national curriculum authorization.

**Responsible institutions:** Individual medical school curriculum committees supported by University of Dschang Faculty of Medicine, Department of Physiotherapy and Physical Medicien (home institution of Cameroon's only two PhD-trained physiotherapists: Dr. Ibrahim Npochinto Moumeni and Dr. Douryan Maurice, both civil servants since 2022); MINESUP coordination.

**Implementation timeline:**

• **Year 1 (2026):** Codify Dschang pilot experience into standardized national module adaptable across diverse institutional contexts; develop faculty training program delivered by Dschang-based experts
• **Year 2 (2027):** Implement in 50 % of medical schools (6–7 institutions) as pilot expansion cohort
• **Year 3 (2028–2030):** Scale to all 13 medical schools; conduct longitudinal impact evaluations tracking knowledge retention, clinical confidence, and post-graduation referral behaviors

**Implementation strategy:** Leverage Dschang model demonstrating significant improvements (knowledge: 41.3 % → 78.7 %; clinical confidence: 23 % → 87 %; sustained application: 94 % at 6-month follow-up) as evidence base; utilize problem-based learning approaches emphasizing immediate clinical applicability per adult learning theory; focus on three discrete competencies (functional assessment, prescription essentials, referral criteria) rather than comprehensive content.

**Estimated costs:** National module adaptation: $15,000–25,000 (9–15 million FCFA); faculty facilitator training (compensation addressing current exploitation: private schools pay physiotherapist educators 3500 FCFA [$5.33 USD] per hour while reducing course hours from 100 h to 20 h for profit): $40,000–60,000 annually (24–36 million FCFA) for adequate compensation and standardized delivery; ongoing delivery costs minimal as integrated into existing courses; potential external funding through WHO Rehabilitation 2030 initiative, Global Rehabilitation Alliance, bilateral development partners. **Total initial investment: $55,000–85,000 USD; Annual delivery: $40,000–60,000 USD.**

**Monitoring indicators:**

• Number of medical schools implementing standardized module (Target: 13/13 by 2030)
• Pre-post knowledge scores (Target: mean increase >30 percentage points across all institutions)
• Six-month post-graduation follow-up surveys (Target: >90 % reporting rehabilitation principles application in clinical practice)
• Faculty compensation adequacy (Target: physiotherapist educators receive minimum $15 USD/h [∼9850 FCFA/h] with standardized course hour requirements)

3. Referral Standardization and Universal Health Coverage Integration.

**Current barrier identified:** Clinical observation revealed only 4.8 % of rehabilitation referrals included adequate clinical context; zero use of functional assessment tools; absence of standardized referral protocols.

**Responsible institutions:** Ministry of Public Health (MINSANTÉ) Clinical Standards Division, in collaboration with Cameroon Association of Physiotherapists (CAPS), hospital quality assurance departments.

**Implementation timeline:**

• **Year 1 (2026):** Develop standardized rehabilitation referral form incorporating functional assessment domains (ICF-based), diagnostic specificity requirements, treatment goal specification; pilot at 10–15 district hospitals
• **Year 2 (2027):** Expand to all regional hospitals; integrate into nascent Universal Health Coverage (UHC) package as prerequisite for rehabilitation service reimbursement
• **Year 3 (2028–2030):** Mandate nationwide implementation; conduct quarterly quality audits

**Strategic UHC integration:** Leverage recent UHC policy development to include rehabilitation services for catastrophic conditions currently excluded: stroke (cerebrovascular accidents), neonatal brachial plexus injuries, cerebral palsy, road traffic accident traumas. These conditions impose devastating financial burden on families yet receive no UHC coverage despite documented high prevalence. **Critical policy recommendation:** MINSANTÉ should amend UHC benefit package to include minimum rehabilitation coverage (12–24 sessions annually) for these four condition categories, with standardized referral form as reimbursement prerequisite ensuring quality control.

**Estimated costs:** Form development and stakeholder validation: $15,000–25,000 (9–15 million FCFA); printing and distribution: $8000–12,000 annually (4.8–7.2 million FCFA); electronic health record integration (where applicable): $40,000–70,000 (24–42 million FCFA, scalable investment); UHC rehabilitation benefit package costing (actuarial analysis): $30,000–50,000 (18–30 million FCFA). **Total: $93,000–157,000 USD.**

**Monitoring indicators:**

• Percentage of rehabilitation referrals using standardized forms (Target: >90 % by 2030)
• Proportion including adequate clinical context (Baseline: 4.8 %; Target: >75 % by 2030)
• Proportion using validated functional assessment tools (Baseline: 0 %; Target: >50 % by 2030)
• UHC rehabilitation claims for stroke, brachial plexus injuries, cerebral palsy, trauma (Target: establish baseline 2027; 50 % coverage increase by 2030)

4. Professional Regulation: Addressing Financial Conflicts of Interest and Establishing Physiotherapist Civil Service Cadre.

**Current barrier identified:** Qualitative interviews documented informal financial arrangements between referring physicians and rehabilitation practitioners; fewer than 5 % of physiotherapists hold civil service positions (salary range: 50,000–100,000 FCFA monthly [$76–152 USD/month]); most operate through hospital space rental arrangements or 50/50 revenue-sharing with hospital directors—practices widely recognized but unregulated.

**Responsible institutions:** National Medical Council (Ordre des Médecins), Ministry of Public Health regulatory divisions, Ministry of Public Service (for civil service recruitment expansion); establishment of professional physiotherapy regulatory body (transitioning CAPS from voluntary association to statutory Order).

**Implementation timeline:**

• **Year 1 (2026):** Develop ethical guidelines on financial relationships in medical referrals with clear conflict-of-interest disclosure requirements; initiate process to establish statutory Ordre des Kinésithérapeutes du Cameroun with regulatory authority comparable to Ordre des Médecins
• **Year 2 (2027):** Launch physician education campaigns on ethical referral practices; implement anonymous patient reporting mechanisms; **Priority action:** MINSANTÉ directive mandating minimum one civil servant physiotherapist per district hospital (currently State recruits 100-330× more nurses than physiotherapists, institutionalizing professional marginalization)
• **Year 3 (2028–2030):** Conduct random audits of referral patterns and financial arrangements; enforce sanctions for documented ethical violations; expand physiotherapist civil service positions to achieve minimum coverage targets

**Implementation strategy:** Current precarious employment status forces physiotherapists into economically compromising arrangements (hospital space rental, revenue-sharing with administrators, kickback agreements with referring physicians) that undermine professional integrity and care quality. Establishing adequate civil service cadre (minimum one physiotherapist per district hospital; expanded staffing at regional/national referral hospitals) creates economically secure positions enabling ethical practice. Civil service physiotherapist salaries should be standardized comparable to other allied health professionals (target: 150,000–250,000 FCFA monthly [$228–380 USD/month], representing 50–150 % increase over current exploitative compensation).

**Estimated costs:** Ethics guideline development: $12,000–20,000 (7.2–12 million FCFA); awareness campaigns: $25,000–40,000 (15–24 million FCFA); establishment of statutory physiotherapy regulatory body: $40,000–60,000 (24–36 million FCFA); **Civil service physiotherapist recruitment (priority investment):** Assuming 150–200 new positions at average 200,000 FCFA monthly ($304 USD): $547,000–730,000 annually (328–438 million FCFA). **Total initial: $77,000–120,000 USD; Annual civil service expansion: $547,000–730,000 USD.**

**Monitoring indicators:**

• Number of documented conflict-of-interest cases investigated (transparency metric; target: systematic investigation of >80 % of reported cases)
• Percentage of physicians reporting awareness of ethical guidelines (Target: >90 % by 2030)
• Number of civil servant physiotherapist positions created (Baseline: <5 % of workforce; Target: minimum 1 per district hospital = ∼180 positions by 2030)
• Physiotherapist employment security and salary adequacy (Target: >50 % holding formal employment with living wage by 2030)

5. Workforce Development and Geographic Distribution: Addressing the “Single Practitioner Syndrome”.

**Current barrier identified:** Study documented extreme geographic maldistribution with single rehabilitation specialist serving entire region (47 % of patients traveling >100 km); nationally, zero physicians trained in Physical and Rehabilitation Medicine (MPR) specialty; only two PhD-trained physiotherapists (both at University of Dschang, both trained abroad—France and Italy respectively).

**Responsible institutions:** Ministry of Public Health workforce planning divisions, Ministry of Higher Education (specialty training program development), professional associations (CAPS, future Ordre des Kinésithérapeutes), international partnership programs (Erasmus+, bilateral cooperation agreements).

**Implementation timeline:**

• **Year 1 (2026):** Conduct comprehensive national rehabilitation workforce census mapping geographic distribution, training levels, population-to-practitioner ratios, identifying priority underserved regions; initiate consultation with Senegal, Côte d'Ivoire, Burkina Faso regarding successful establishment of domestic MPR specialty training programs
• **Year 2 (2027):** Develop rural/underserved area incentive schemes for rehabilitation professionals (housing allowances, salary supplements 25–50 % above urban rates, student loan forgiveness programs, accelerated career progression); establish scholarship programs for physiotherapist advanced training (Masters, PhD) through partnerships with francophone universities (France, Belgium, Senegal); **Priority action:** Initiate feasibility assessment for establishing domestic MPR residency training program at major teaching hospitals (University Teaching Hospitals Yaoundé, Douala, potentially Dschang given existing expertise concentration)
• **Year 3 (2028–2030):** Implement incentive schemes to attract rehabilitation professionals to underserved regions; expand advanced training opportunities increasing domestic PhD-trained physiotherapist faculty from current two to minimum 10–15 by 2030; develop domestic postgraduate medical training capacity in rehabilitation medicine

**Implementation strategy:** Benchmark successful francophone African models: Senegal, Côte d'Ivoire, Burkina Faso established domestic MPR specialty training, offer scholarships for advanced physiotherapy training abroad, maintain adequate professional salaries enabling retention. Cameroon currently depends entirely on foreign-trained rehabilitation professionals; the only two PhD-trained physiotherapists both completed training abroad (France via Sorbonne University, Italy via University of Rome Tor Vergata) and returned as civil servants despite better remuneration opportunities abroad, demonstrating potential for retention if adequate positions exist. Geographic maldistribution stems not from professionals' unwillingness to serve underserved areas but from absence of formal positions and infrastructure.

**Estimated costs:** National workforce assessment: $35,000–55,000 (21–33 million FCFA); rural incentive schemes: $7500–12,000 per practitioner annually (4.5–7.2 million FCFA), estimated 25–40 practitioners = $187,500–480,000 annually (112–288 million FCFA); advanced training scholarships: $25,000–40,000 per scholar annually (15–24 million FCFA), 5–8 scholars = $125,000–320,000 annually (75–192 million FCFA); domestic MPR residency program establishment: $250,000–400,000 initial investment (150–240 million FCFA), $100,000–150,000 annual operating costs (60–90 million FCFA). **Total initial: $597,500-1,255,000 USD; Annual: $412,500–950,000 USD.**

**Monitoring indicators:**

• Rehabilitation professional-to-population ratios by region (Target: reduce inter-regional coefficient of variation by 50 % by 2030)
• Percentage of population living within 50 km of rehabilitation services (Target: >70 % by 2030, up from current 53 %)
• Average patient travel distance for rehabilitation consultations (Baseline: 47 % travel >100 km; Target: <30 % by 2030)
• Number of PhD-trained physiotherapist faculty (Baseline: 2; Target: 10–15 by 2030)
• Establishment of domestic MPR residency training program (Target: first cohort enrollment by 2028–2029 academic year)

### 8.2 Overall implementation coordination and financing

These recommendations align directly with WHO Regional Strategy operational objectives, particularly Objective 3 (workforce capacity building) and Objective 2 (service integration across health system levels). Successful implementation requires:

**Governance structure:** Establish national multisectoral Rehabilitation Integration Steering Committee chaired by MINSANTÉ, including representatives from MINESUP, Ministry of Finance, Council of Medical Deans, University of Dschang Faculty of Medicine, CAPS, hospital administrators, patient advocacy organizations, WHO AFRO technical advisor. Quarterly steering committee meetings monitoring implementation progress against established indicators.

**Financing strategy:**

• **Total estimated 5-year implementation cost: $2.3–4.1 million USD** (approximately 1.4–2.5 billion FCFA)
• **Domestic government allocation:** 30–40 % from national health budget and higher education budget reallocation = $690,000-1,640,000 USD
• **External development partner support:** 60–70 % from WHO Rehabilitation 2030 initiative, World Bank Universal Health Coverage programs, bilateral cooperation (French Development Agency/AFD, Belgian Development Cooperation), Global Rehabilitation Alliance = $1,380,000-2,870,000 USD
• **Cost-effectiveness consideration:** Investment represents <0.1 % of national health expenditure yet addresses documented service gap affecting millions; successful francophone African comparators (Senegal, Côte d'Ivoire) achieved similar reforms with comparable investments

**Accountability mechanisms:** Annual progress reports presented to National Assembly Health Committee; bi-annual stakeholder review conferences assessing implementation barriers and adaptations; integration of rehabilitation workforce and service indicators into national Health Management Information System (HMIS); public dashboard tracking progress against WHO Regional Strategy 2025–2035 targets.

These operational pathways transform high-level policy recommendations into actionable interventions with clear institutional accountability, realistic resource mobilization strategies, and measurable outcomes. By addressing the documented institutional mechanisms excluding rehabilitation from medical education—coercive isomorphism (absence of ministerial guidelines), normative isomorphism (faculty knowledge deficits), and mimetic isomorphism (following inadequate international models)—while simultaneously strengthening professional regulation, workforce development, and financing mechanisms, Cameroon can establish sustainable rehabilitation system integration directly supporting WHO Regional Strategy implementation and achieving meaningful population health improvements for conditions currently causing catastrophic household expenditure and long-term disability burden.

## 9 Limitations and future

### 9.1 Limitations

Several limitations must be acknowledged in this study, with implications for both internal and external validity.

First, our single-site clinical observation at Bafoussam Regional Hospital may not fully represent rehabilitation referral patterns across all regions of Cameroon or the broader Central African context. Although the wide geographic distribution of patient origins (47 % traveling over 100 km) suggests our findings capture referral patterns from a broad catchment area, clinic-level idiosyncrasies—including local resource availability, institutional culture, and the particular characteristics of the single rehabilitation specialist practicing at this site—may limit generalizability [41]. The absence of comprehensive epidemiological data on rehabilitation needs and service utilization patterns in Cameroon further constrains our ability to situate these findings within national health system performance metrics [35]. This data deficit reflects broader challenges in African health information systems, where rehabilitation services remain poorly documented in national health statistics and disease surveillance systems [42]. Multicenter observational studies across diverse clinical settings (urban/rural, teaching/non-teaching hospitals, different francophone African countries) would strengthen external validity and clarify which findings represent systemic patterns versus site-specific phenomena.

Second, our qualitative sample of medical education leaders (*n* = 12) may reflect self-selection bias, as participants who agreed to interviews might hold more progressive views on rehabilitation integration than non-participants. This could result in underestimation of institutional resistance to curricular reform or overestimation of readiness for change. Conversely, those most resistant to rehabilitation integration may have been more willing to articulate concerns, potentially overrepresenting barriers [43]. Future research employing stratified random sampling, anonymous survey methodologies, or administrative mandate for participation could better capture the full spectrum of institutional perspectives and reduce volunteer bias.

Third, the clinical observation component faces potential Hawthorne effects, whereby the rehabilitation practitioner may have modified prescribing behaviors due to awareness of being observed [44]. While we employed prolonged engagement over 24 months and integrated observation into routine clinical workflows to minimize reactivity, we cannot entirely exclude the possibility that documented prescription quality (4.8 % with adequate clinical context, 0 % with functional assessment tools) represents an improvement over truly unobserved practice. The magnitude of poor prescription quality observed suggests that even if Hawthorne effects existed, baseline practice patterns likely remain substantially deficient. Nevertheless, covert observation designs—though ethically complex—or administrative database analyses might provide more naturalistic estimates of prescription quality.

Fourth, our assessment of the Dschang educational intervention evaluated immediate outcomes and six-month follow-up rather than conducting a prospective study that assesses long-term retention and application of knowledge over time. While the six-month follow-up (response rate 72 %, *n* = 63) provided preliminary evidence of sustained behavioral change (94 % reported using rehabilitation principles in clinical rotations), future research should employ longitudinal designs tracking medical students from curriculum exposure through residency training and early practice years to evaluate the durability of knowledge gains and their translation into actual referral behaviors in independent practice settings [45].

Fifth, while we documented the existence of financial incentives in referral patterns through qualitative interviews, we were unable to quantify their prevalence systematically due to the sensitive nature of this information. The absence of regulatory oversight and lack of transparent referral tracking systems in the Cameroonian health system precluded objective measurement of informal payment practices. More robust methodologies—potentially including anonymous reporting systems, administrative claims data analysis, or audit studies using standardized patient scenarios—could better characterize the prevalence and magnitude of these practices and their impact on care access.

Sixth, and critically, we were unable to track patient-level clinical outcomes resulting from inadequate physician referral practices. The documented poor prescription quality (only 4.8 % with adequate clinical context, zero use of standardized functional assessment tools) almost certainly translates into suboptimal patient functional outcomes, delayed access to appropriate care, increased out-of-pocket expenditures due to inappropriate referrals, and greater long-term disability burden. However, our study design did not permit direct quantification of these downstream health consequences. Prospective cohort studies linking physician referral patterns to patient functional status trajectories, quality-of-life measures, rehabilitation service utilization, time-to-treatment intervals, and economic burden would strengthen causal inferences regarding the population health impacts of educational deficits in rehabilitation training.

### 9.2 Future research directions

Future research should address these limitations through complementary methodological approaches:

1. Longitudinal cohort studies tracking the impact of rehabilitation education on physician referral patterns over 3–5 years, with direct measurement of patient functional outcomes and rehabilitation service utilization
2. Implementation science approaches evaluating the transferability of the proposed curriculum model to different Central African contexts, with attention to context-specific barriers and facilitators
3. Cost-effectiveness analyses of curriculum integration compared to standalone training initiatives, incorporating both health system costs and patient out-of-pocket expenditures
4. Patient-centered outcomes research linking educational interventions to functional improvements in rehabilitation access and quality, including measures of equity in service delivery across geographic and socioeconomic gradients
5. Development of national rehabilitation epidemiological databases in Cameroon and comparable Central African settings to establish baseline metrics for monitoring health system strengthening efforts aligned with WHO Regional Strategy implementation
6. Multi-country comparative studies examining how different historical, political, and educational governance structures shape rehabilitation integration patterns across francophone African medical education systems

## 10 Conclusion

Cameroon's exclusion of rehabilitation from medical curricula stems from entrenched institutional mechanisms—absent accreditation requirements, professional hierarchies that devalue rehabilitation, and uncritical replication of inadequate curricula. These mechanisms perpetuate professional marginalization and create barriers to care. The resulting “Single Practitioner Syndrome,” where isolated specialists serve disproportionate catchments, represents a previously unrecognized phenomenon with implications for health systems across resource-limited settings. This syndrome creates structural inefficiencies (47 % of patients traveling >100 km), distorts referral pathways (only 4.8 % of prescriptions include adequate clinical context), generates informal economies (revenue-sharing arrangements between prescribers and practitioners), and degrades care quality through professional isolation.

Even brief educational interventions can improve rehabilitation knowledge and referral practices substantially. The 4-h module at University of Dschang increased knowledge from 41 % to 79 % and referral confidence from 23 % to 87 %, with 94 % of students applying these principles six months later. This demonstrates that curriculum reforms targeting core competencies—functional assessment, prescription essentials, appropriate referral criteria—can strengthen rehabilitation integration without extensive resources.

Implementing the WHO Regional Strategy to Strengthen Rehabilitation in Health Systems 2025–2035 requires addressing these foundational educational barriers. Our policy recommendations provide actionable pathways: establishing rehabilitation competencies in national accreditation standards during the 2025–2026 curriculum revision; scaling the Dschang model across all 13 medical schools; integrating rehabilitation services (stroke, neonatal brachial plexus injuries, cerebral palsy, trauma) into Cameroon's new Universal Health Coverage package; recruiting civil servant physiotherapists (minimum one per district hospital) to eliminate exploitative employment practices; and expanding advanced training to address the current workforce crisis (zero physicians trained in Physical and Rehabilitation Medicine; only two PhD-trained physiotherapists nationally). Estimated five-year implementation cost of $2.3–4.1 million represents modest investment relative to population health gains, with financing from domestic allocation (30–40 %) and external partners (60–70 %) including WHO, World Bank, and bilateral cooperation.

This study provides both warning and roadmap. Medical education's exclusion of rehabilitation creates cascading system failures—physician knowledge deficits, inadequate referrals, workforce undersupply, service monopolization, structural inefficiencies, and population-level inequities. Yet practical, resource-conscious interventions can break this cycle. By targeting the institutional mechanisms maintaining exclusion, policymakers can create sustainable pathways to improved rehabilitation access and better functional outcomes for patients across Central Africa.

## Ethical approval and consent

This observational educational research was conducted in accordance with the ethical principles outlined in the Declaration of Helsinki (2013 revision) for research involving human participants.

**Institutional interviews**

Academic leaders participated voluntarily in semi-structured interviews after being fully informed of the research purpose, procedures, and their right to withdraw at any time. Verbal informed consent was obtained prior to each interview. All interview data were completely anonymized with no identifying information retained. Institutional affiliations are reported only at the aggregate level (e.g., “Cameroonian medical schools”) to protect participant confidentiality.

**Clinical observations**

Clinical observations utilized routine clinical documentation practices at Bafoussam Regional Hospital. Patient data were recorded as part of standard care documentation with strict confidentiality protections. All patient data presented in this manuscript have been anonymized with no personally identifiable information disclosed. Geographic origins are reported at the city level only, with no specific patient identifiers.

The observational nature of this study, utilizing existing clinical documentation and institutional discussions without experimental interventions, falls under the category of quality improvement research within standard healthcare delivery frameworks.

Given the observational nature and complete anonymization of all data, formal institutional review board approval was not required under Cameroonian research ethics guidelines. However, all research activities adhered to principles of beneficence, non-maleficence, autonomy, and justice throughout the study.

## CRediT authorship contribution statement

**Ibrahim Npochinto Moumeni:** Writing – review & editing, Writing – original draft, Visualization, Validation, Supervision, Software, Resources, Project administration, Methodology, Investigation, Formal analysis, Data curation, Conceptualization. **France Mourey:** Validation, Supervision, Methodology, Formal analysis. **Faustin Atemkeng Tsatedem:** Visualization, Supervision, Methodology, Investigation, Formal analysis. **Kossi Oyene:** Software, Resources, Methodology. **Yacouba Njankouo Mapoure:** Writing – review & editing, Writing – original draft, Supervision, Project administration, Methodology.

## Funding statement

This research received no specific grant from any funding agency in the public, commercial, or not-for-profit sectors. All research activities were self-funded by the lead author through personal resources.

## Declaration of competing interest

The authors declare no financial or non-financial competing interests relevant to this research. None of the authors have financial relationships with any organizations that might have an interest in the submitted work, no patents or copyrights related to this work, and no other relationships or activities that could appear to have influenced the submitted work.

The authors affirm that this manuscript represents original work that has not been published previously and is not under consideration for publication elsewhere.

## Data Availability

The datasets generated and analyzed during the current study are not publicly available due to privacy and confidentiality considerations (institutional interviews and patient clinical observations), but anonymized aggregated data supporting the findings are available from the corresponding author upon reasonable request and subject to appropriate data sharing agreements that maintain participant confidentiality. Interview transcripts have been permanently anonymized with all identifying information removed. Summary thematic analyses can be shared to support research transparency while protecting participant identities.
